# SCCmecExtractor: a tool for extracting staphylococcal cassette chromosome elements from whole-genome sequences

**DOI:** 10.1099/mgen.0.001809

**Published:** 2026-11-05

**Authors:** Alison C. MacFadyen

**Affiliations:** 1 The Sainsbury Laboratory, Norwich Research Park, Norwich, NR4 7UH, UK; 2 Centre for Microbial Interactions, Norwich Research Park, Norwich NR4 7UG, UK; 3 University of East Anglia, Norwich Research Park, Norwich, Norfolk, NR4 7TJ, UK

**Keywords:** antimicrobial resistance, bioinformatics, methicillin-resistant *Staphylococcus aureus* (MRSA), mobile genetic elements, SCC*mec*, *Staphylococcus*

## Abstract

Staphylococcal cassette chromosome (SCC) elements are mobile genetic elements that integrate at the *rlmH* gene and are predominantly responsible for methicillin resistance in staphylococci. Although staphylococcal cassette chromosome *mec* (SCC*mec*) typing tools exist, none can extract the element sequence itself or explicitly classify SCC elements that lack methicillin resistance genes. Here, we present SCC*mec*Extractor, a lightweight Python toolkit that identifies SCC element boundaries through degenerate attachment site (*att*) pattern matching, extracts complete elements from whole-genome assemblies and characterizes their *mec* and *ccr* gene content, providing SCC*mec* type classification where applicable. Benchmarking on 7,297 genomes spanning 70 species across *Staphylococcus* and *Mammaliicoccus* demonstrated 94.8% SCC*mec* type concordance with the sccmec tool [1] on 1,454 *Staphylococcus aureus* genomes, with all eight disagreements involving elements with multiple *ccr* complexes. The tool extracted 1,562 SCC elements from 1,454 *S*. *aureus*, 5,295 non-*aureus Staphylococcus* and 548 *Mammaliicoccus* genomes, achieving effective extraction rates (excluding assembly-limited genomes and those lacking valid *ccr* pairs) of 87.3% for *S. aureus*, 58.8% for non-*aureus Staphylococcus* and 61.9% for *Mammaliicoccus*. A hybrid detection module classifies extracted elements by similarity to 32 SCC*mec* type references, distinguishing canonical single-type elements, hybrid elements showing recombination-derived similarity to multiple types and multi-*ccr* elements consistent with structural tandem/nested elements. Notably, 616 of the 1,562 extracted elements (39.4%) were non-*mec* SCC elements lacking methicillin resistance genes, a class of mobile element often overlooked. Non-*mec* SCC prevalence increased from 12.2% in *S. aureus* to 55.6% in non-*aureus Staphylococcus* and 76.0% in *Mammaliicoccus*, revealing a substantial reservoir of SCC diversity beyond methicillin resistance. SCC*mec*Extractor is freely available via PyPI, Docker and Singularity under an MIT licence.

**Impact Statement**

Staphylococcal cassette chromosome (SCC) elements are mobile genetic elements responsible for methicillin resistance in staphylococci and are central to methicillin-resistant *Staphylococcus aureus* epidemiology. Existing tools focus on typing SCC*mec* (staphylococcal cassette chromosome *mec*) from assemblies but cannot extract the element itself, limiting our ability to comprehensively monitor and examine these elements. SCC*mec*Extractor is a lightweight, portable tool that detects the attachment sites required by SCC elements to integrate into the genome, extracts SCC elements regardless of *mec* gene carriage, characterizes their gene content to assign SCC*mec* type (I-XV), where applicable, and performs hybrid element detection. Applied across 7,297 genomes spanning two genera, we demonstrate that non-*mec* SCC elements are the dominant SCC class outside *S. aureus*, a finding enabled by systematic extraction and classification of SCC elements regardless of *mec* gene content. SCC*mec*Extractor provides the research community with an accessible, confidence-first approach (based on biology) to SCC element analysis across all staphylococci and mammaliicocci species.

## Data Summary

The code for this pipeline is available at: https://github.com/AlisonMacFadyen/SCCmecExtractor, with a Docker image available at: https://hub.docker.com/repository/docker/alisonmacfadyen/sccmecextractor and PyPi package at: https://pypi.org/project/sccmecextractor/. All reference databases are bundled with the tool. Benchmarking genome accessions: 1,454 *Staphylococcus aureus*, 5,295 non-aureus *Staphylococcus* and 548 *Mammaliicoccus* genomes from NCBI. A complete list of genome accessions is provided as supplementary data (Table S1). Extracted staphylococcal cassette chromosome elements can be obtained from Zenodo: https://zenodo.org/records/19355206

## Introduction

Methicillin-resistant *Staphylococcus aureus* (MRSA) is an important human and animal pathogen [2], with MRSA being a major cause of human death occurring due to antimicrobial resistance worldwide [3]. Acquisition of *mec* genes, most commonly *mecA*, confers resistance to methicillin in *staphylococcus* species, by encoding an alternative penicillin-binding protein (PBP2a) with low affinity for β-lactam antibiotics [4, 5]. These *mec* genes are carried on staphylococcal cassette chromosome *mec* (SCC*mec*), a mobile genetic element that integrates site-specifically into the chromosome at the 3′ end of the *rlmH* gene (formerly *orfX*) [6, 7].

Integration and excision of SCC*mec* is achieved via element carrying large serine recombinases, CcrAB or CcrC, which identify specific DNA attachment sites (*att*) [8]. For integration, a pair of *att* sites, *attB* on the chromosome and *attSCC* on the circularized SCC*mec* element, are required. After integration, two *att* sites are generated flanking the SCC*mec* element: *attR* (*rlmH*-proximal) and *attL* (distally located) [8]. Each *att* site comprises two half-sites flanking a central GA dinucleotide where strand exchange occurs; these half-sites form degenerate inverted repeats [9]. The half-sites are shared between *att* sites: three are similar to one another and derive from the SCC*mec* element, while the fourth overlaps the *rlmH* coding frame and is found within *attB* and *attL* [9]. All four full *att* sites contain a conserved 8 bp core motif (TATCATAA), including a central cytosine thought to be essential for recombination [10]. Our *attR* search patterns capture the region spanning 3 bp upstream to 12 bp downstream of this core motif, whereas *attL* patterns target the SCC-derived half-site upstream of the core, including the variable bridge region that reflects the diversity of integrated elements.

SCC*mec* elements carry two essential genetic components: the *mec* gene complex, comprising the *mec* gene, and in some cases, the regulatory elements (*mecR1*, *mecI*), and the *ccr* gene complex [11], encoding the large serine recombinase (*ccrA*/*ccrB* pairs or *ccrC*) [12]. The remainder of the element consists of joining (J) regions that are varied and can be highly diverse [13]; these regions are also the basis of defining SCC*mec* subtypes [12]. The International Working Group on the Classification of Staphylococcal Cassette Chromosome Elements (IWG-SCC) classifies SCC*mec* types based on the combination of *ccr* gene complex type (determined by *ccr* allotype) and *mec* gene complex class (determined by regulatory genes and insertion sequences [ISs]) [14]. To date, 15 SCC*mec* types (I–XV) have been defined for *S. aureus* [12, 15, 16].

SCC elements are not limited to SCC*mec*, with staphylococci carrying a diversity of SCC elements which lack methicillin resistance genes. These non-*mec* SCC elements retain the hallmarks of SCC, the *att* site boundaries, *ccr* recombinases and integration at *rlmH* but carry a variety of different genes, e.g. mercury resistance (SCC*Hg*), capsule biosynthesis (SCC*cap1*) etc [17–19]. Despite their potential roles in bacterial adaptation and as vehicles for horizontal gene transfer, non-*mec* SCC elements are largely overlooked because existing tools focus on methicillin resistance.

Throughout this manuscript, we use SCC to refer to all SCC elements regardless of *mec* gene carriage, SCC*mec* for SCC elements carrying *mec* genes and non-*mec* SCC for SCC elements lacking *mec* genes.

Several bioinformatic tools have been developed for SCC*mec* analysis. The sccmec tool [1] uses a targets-and-regions blast approach to assign formal SCC*mec* types I–XV from assembly data. However, it does not detect *att* sites or extract element sequences and although it identifies the presence of *ccr* genes, it does not specifically name non-*mec* SCC elements. SCC*mec*Finder, developed by the Center for Genomic Epidemiology (CGE), combines blast and k-mer approaches for typing, but the standalone tool requires Python 2.7 and legacy blast, making it effectively non-functional; its web server is functional but limited to types I–XI [20]. Similar to sccmec, SCC*mec*Finder identifies the presence of *ccr* genes in the absence of *mec* and will state that the organism is most likely methicillin-sensitive *S. aureus* but does not formally identify the element as a non-*mec* SCC. Finally, SCC*mec*_CLA [21] performs extraction of SCC*mec* elements. This is achieved by searching the distal *att* site using the 8 bp core motif (TATCATA[AT]), with blast validation against known references, then assigns the start of the element as the 3′ region of *rlmH*, without explicit *att* site detection. Extracted SCC elements are typed (I–XI) through gene synteny for *mec* class and *ccr* allotype assignment, which is supplemented with k-nearest-neighbour subtype prediction using Mash distances to 267 reference cassettes. Although a sophisticated approach, it requires all three core components (*rlmH*, *mecA* and *ccr*) and therefore cannot detect non-*mec* SCC elements, is limited to *S. aureus* by its single-species *rlmH* reference and has undocumented dependencies that prevent reproducible installation.

A gap therefore exists for a lightweight, cross-platform tool that can: (i) detect SCC element boundaries through explicit *att* site identification; (ii) extract complete element sequences; (iii) characterize gene content at the allotype level; (iv) detect non-*mec* SCC elements; and (v) operate across staphylococcal and mammaliicoccal species. SCC*mec*Extractor addresses this gap.

## Implementation

Integration of SCC elements requires two key elements, the presence of recombinase, to carry out the integration, and attachment sites for recognition of the recombination site. Therefore, a key approach of SCC*mec*Extractor is to identify those attachment, or *att* sites, then determine if the genes encoding the recombinases, *ccr* genes, are present between the *att* site location. Thus, incorporating our biological understanding of how these elements integrate to identify them and extract their sequences from whole-genome sequence (WGS) data. The primary functions of SCC*mec*Extractor are: sccmec-locate-att, sccmec-extract, sccmec-type, sccmec-hybrid, sccmec-report and sccmec-pipeline, as outlined in Fig. 1. Input files can either be FASTA/FNA only or FASTA/FNA with a GFF3 annotation file.

**Fig. 1.**
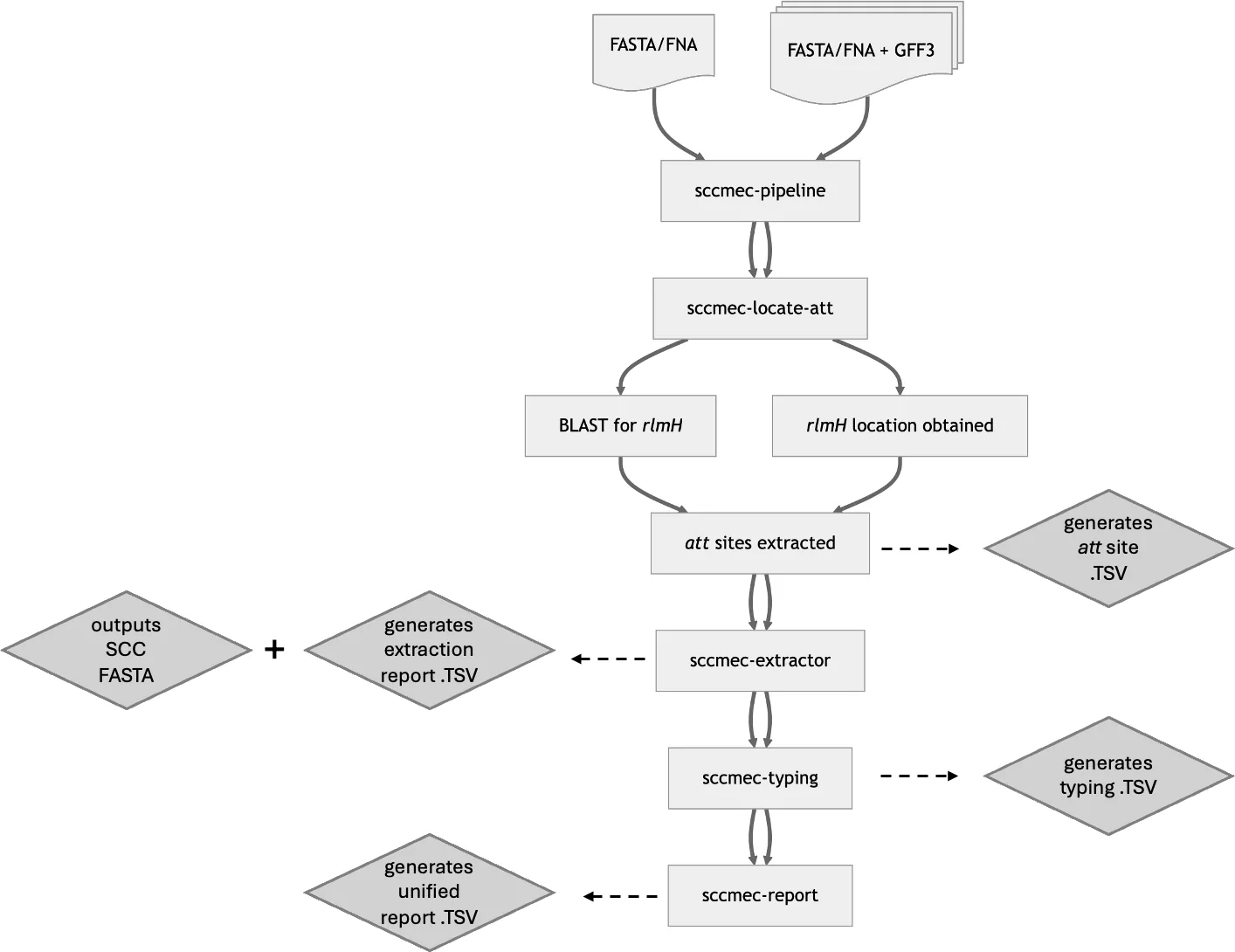
Schematic overview of the SCC*mec*Extractor pipeline showing the six command-line tools and their data flow. Input genomes (FASTA/FNA with optional GFF3) are processed through: (1) sccmec-locate-att for attachment site detection with *rlmH* validation; (2) sccmec-extract for SCC element extraction; (3) sccmec-type for blast-based gene-level typing of *mec* and *ccr* allotypes, and SCC*mec* type (I–XV) assignment; (4) sccmec-hybrid for determining if extracted SCC element are hybrids of SCC*mec* types (I–XV); (5) sccmec-report for merging extraction and typing results into a unified and summary report. The master pipeline sccmec-pipeline orchestrates all steps in a single command. Arrows indicate data flow between modules; dashed arrows show the files/outputs produced by that step/tool.

Each module produces specific outputs that users can review:

- sccmec-locate-att: att_sites/ – a directory containing per-genome *att* site information
- sccmec-extract: sccmec/– a directory with extracted element FASTA files
- sccmec-extract: ambiguous_att_sites.tsv – detailed *att* site information for non-extracted genomes
- sccmec-extract: extraction_report.tsv – extraction report (standalone mode; merged into unified report when using the pipeline) containing comprehensive metadata associated with extraction success or failure
- sccmec-type: typing.tsv – if used standalone, produces a summary SCC*mec* typing output
- sccmec-hybrid: hybrid_summary.tsv – reference comparison results for extracted elements, used to detect hybrid elements
- sccmec-hybrid: hybrid_detail/ – (optional with -d flag) detailed reference comparison results for extracted elements, one per genome
- sccmec-report: sccmec_summary.tsv – start here. One row per genome with key results.
- sccmec-report: sccmec_unified_report.tsv – full diagnostic detail for all genomes (all columns).

### Attachment site detection – sccmec-locate-att

As integration of SCC elements requires identification of *attB* and recombination with *attSCC*, resulting in the generation of *attR* and *attL* flanking the region, the first step in the workflow is to locate these *att* sites. Detection of these sites is achieved using 20 regular expression patterns, 4 forward and 4 reverse-complement for *attR* variants, with 6 forward and 6 reverse-complement patterns for *attL* variants, capturing species-specific bridge sequence diversity (Table S2). All patterns were validated by cross-referencing detected *att* site coordinates against GFF3 gene annotations across all 7,297 benchmarking genomes, confirming that *attL* sites fall in intergenic regions, while *attR* sites fall within *rlmH* as expected. Further to this, the majority of *att* sites contained the GA dinucleotide, located 6 bp upstream of the central 8 bp core motif, which is hypothesized to be where DNA strand exchange occurs during excision/integration [9]. The only exception was *attL14*, which carries a variant core motif (TATCCTAA) and an AA dinucleotide in place of GA. Despite this sequence divergence, *attL14* was retained as a valid pattern because it is exclusively associated with *ccrA2*/*ccrB2* at standard identity thresholds, paired with canonical *attR*, is restricted to *S. aureus* clinical isolates and consistently defines a distal SCC*mec* boundary flanked by IS21 elements, consistent with a genuine, if divergent, recombination product.

The variance within the patterns accounts for diversity across species. For example, the core *attR* pattern GC[AGCT]TATCA[TC]AA[GA]TGATGCGGTTT incorporates four potential nucleotides at position 4 as this accounts for *S. aureus* variants identified via systematic analysis of 1,454 complete genomes. For *attL,* the patterns include conserved flanking motifs of 5 and 6 bp (CATCA.GATAAG); these motifs are bridged by variable-length sequences ranging from 5 to 15 bp.

Once the *att* sites have been detected, further checks are undertaken to confirm that *attR* is identified at the expected location, e.g. within 100 bp of *rlmH*. Two modes are available for *rlmH* detection: GFF3-based (parsing the gene annotations) or blast-based (querying a bundled 70-species *rlmH* reference database). The blast mode enables FASTA-only input, removing the requirement for genome annotation, with users able to provide their own reference *rlmH* gene.

Results are output as a per-genome TSV file recording all detected *att* sites with their positions, patterns and strand orientation, enabling further exploration of the *att* site patterns and genomic location.

### SCC element extraction – sccmec-extract

The extraction module receives *att* site locations and applies a series of biological validation steps to determine whether a complete SCC element can be extracted (Fig. 2).

**Fig. 2.**
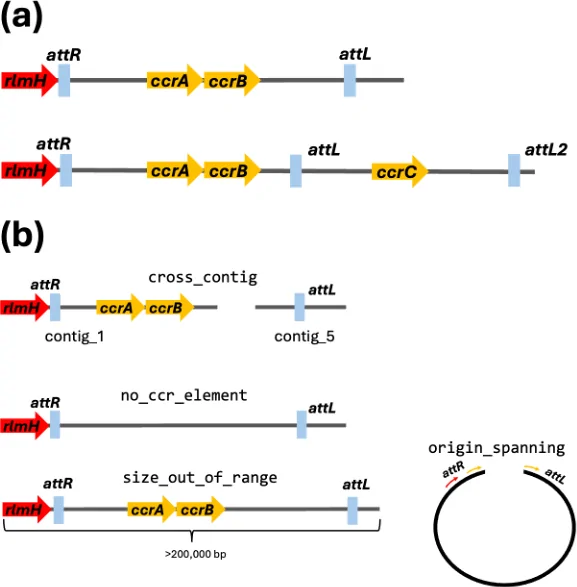
Overview extraction requirements. After obtaining valid *att* sites from sccmec-locate-att, sccmec-extract performs additional checks to determine if an SCC element can be extracted. (**a)** Successful SCC element extraction with *att* sites located on the same contig, *ccr* genes present between the *att* sites and *attR* located within proximity to *rlmH*. Note that composite elements are also extracted using the *attL* furthest from *attR* as the outer limit. (**b)** SCC element failure scenarios: *attR* and *attL* are located on different contigs (cross_contig); *ccr* genes are missing between *attR/rlmH* and *attL* (no_ccr_element); element size exceeds the threshold, e.g. 200,000 bp for a single element, 250,000 bp for composite elements (size_out_of_range); and there is also a cross-origin check for *att* sites that span the chromosome boundary (origin_spanning).

#### Same-contig requirement

Both *attR* and *attL* must reside on the same contig. For draft assemblies where SCC elements may span contig boundaries, the tool reports the failure reason (cross_contig) rather than attempting contig concatenation, prioritizing extraction confidence over completeness.

#### *ccr* gene check

After identifying a valid *att* site pair, a blast search confirms the presence of *ccr* genes between the *att* sites. Elements with *att* site pairs but no *ccr* genes are classified as no_ccr_element (potentially staphylococcal cassette islands, SCI) and recorded in the extraction report but not extracted as FASTA.

#### Size filter

Extracted elements must fall between 1,000 bp and 200,000 bp for single elements, with composite size increased to 250,000 bp for the upper limit. This rejects pattern-overlap artefacts and genome-spanning spurious matches.

#### Composite element detection

When multiple *att* site pairs are detected on the same contig, the tool identifies composite SCC element structures. With the --compositeflag, extraction extends to the outermost *att* site boundary and reports both inner and outer element coordinates, with *ccr* gene checks required within the outer limit to be considered genuine.

#### Fallback extraction

When only *attL* is detected (no *attR*), a fallback pathway infers the right boundary from the *rlmH* gene position. This requires: (i) *rlmH* on the same contig as *attL*; (ii) *attL* within 120 kbp of *rlmH*; and (iii) *ccr* genes between *attL* and *rlmH*. This is also reported as fallback_extracted alerting users that this path was taken for extraction.

#### Origin-spanning detection

Circular chromosomes linearized at the SCC element locus produce *att* sites at opposite ends of the sequence, yielding apparent element sizes covering most of the chromosome. When the calculated element size exceeds the maximum but the wrap-around distance (contig length minus element size) falls within a plausible range, the genome is flagged as origin_spanning with coordinates for manual extraction.

#### Extraction diagnostics

Every genome receives an extraction status and, for failures, a categorized reason: right_only, left_only, cross_contig, no_sites, size_out_of_range, no_ccr_element or origin_spanning. This transparent reporting enables users to understand why extraction failed and to assess whether a more contiguous assembly might resolve the failure or if these are non-addressable failures. Extraction performance is reported as two complementary metrics: a *ccr*-positive extraction rate (extracted elements as a proportion of all *ccr*-positive genomes) and an effective extraction rate that excludes genomes where extraction is impossible regardless of tool capability. Specifically, assembly-limited genomes (where *att* sites fall on different contigs, span the sequence origin or where *rlmH* is on a separate contig from *attL*) and genomes with unpaired *ccr* genes (a lone *ccrA* or *ccrB* without a functional recombinase pair). The effective rate measures tool performance on genomes where extraction is theoretically achievable.

When running the sccmec-extract module as standalone, along with the extracted SCC element FASTA sequences is an 18-column TSV extraction report with comprehensive metadata including element coordinates, size, composite status, contig edge proximity and diagnostic notes. For both the sccmec-pipeline and sccmec-extract approaches, there is also a separate ambiguous_att_sites.tsv file, which collates all *ccr*-positive, non-extracted genomes with their failure reason, detected *att* site positions, *rlmH* contig, element size and explanatory notes. It also includes information on genomes with *attR* and *attL* detected but no *ccr* genes, as these could contain novel recombinases. This file enables users to investigate extraction failures, for example, identifying cross-contig cases that would be resolved with improved assemblies, examining *att* site pairs that lack *ccr* genes (SCI elements) or locating origin-spanning elements whose coordinates are provided for manual extraction.

### Gene level typing – sccmec-type

Gene-level typing is performed via blast against bundled reference databases containing 9 *mec* allotypes (*mecA*, *mecA1*, *mecA2*, *mecB*, *mecC*, *mecC1*, *mecC2*, *mecC3*, *mecD*), 31 *ccr* allotypes (*ccrA1-ccrA14*, *ccrB1-ccrB12*, *ccrC1-ccrC5*) and *mec* class associated genes (*mecR1, mecI, IS431, IS1272, blaZ*) (Table 1).

**Table 1.** *mec*, *ccr* and *mec* class associated gene references for gene level typing analysis. SCC*mec*Extractor uses bundled reference databases for *mec*, *ccr* and associated *mec* complex genes detection via blast. The table lists all reference allotypes with their GenBank accessions and classification thresholds

Reference gene	Accession no.	Nucleotide identity (%)	Nucleotide coverage (%)
*mecA*	KC243783.1	≥95	≥90
*mecA1*	Y13094.1 : 2045–4045	≥95	≥90
*mecA2*	AM048810.2	≥95	≥90
*mecB*	AB498758.1 : 3738–5762	≥95	≥90
*mecC*	FR821779.1 : 35681–37678	≥95	≥90
*mecC1*	HE993884.1 : 12033–14029	≥95	≥90
*mecC2*	KF955540.2 : 436–2433	≥95	≥90
*mecC3*	MH155596 : 119542–121539	≥95	≥90
*mecD*	KY013611.1 : 15438–17474	≥95	≥90
*ccrA1*	AB063171.1 : 12055–13404	≥84.5	≥90
*ccrA2*	AB063172.2 : 9888–11237	≥84.5	≥90
*ccrA3*	AB014436.1 : 134–1480	≥84.5	≥90
*ccrA4*	AF411935.3 : 7849–9210	≥84.5	≥90
*ccrA5*	AM904731.1 : 4627–5976	≥84.5	≥90
*ccrA6*	MN220716.1:c48983-47634	≥84.5	≥90
*ccrA7*	AB587080.1 : 1–1353	≥84.5	≥90
*ccrA8*	OL880246.1 : 172–1572	≥84.5	≥90
*ccrA9*	CP065792.1:c612238-610889	≥84.5	≥90
*ccrA10*	CP069486.1:c972238-970889	≥84.5	≥90
*ccrA11*	CP063367.1:c47706-46357	≥84.5	≥90
*ccrA12*	CP016760.1:c2324024-2322675	≥84.5	≥90
*ccrA13*	CP065795.1 : 1089818–1091167	≥84.5	≥90
*ccrA14*	LT906460.1:c47750-46416	≥84.5	≥90
*ccrB1*	AB063171.1 : 13426–15054	≥84.5	≥90
*ccrB2*	AB063172.2 : 11259–12887	≥84.5	≥90
*ccrB3*	AB014436.1 : 1501–3129	≥84.5	≥90
*ccrB4*	AF411935.3 : 9207–10535	≥84.5	≥90
*ccrB5*	AM904731.1 : 5997–7625	≥84.5	≥90
*ccrB6*	AP008934.1:c43044-41416	≥84.5	≥90
*ccrB7*	AB353724.1:c17096-15468	≥84.5	≥90
*ccrB8*	AB498756.1:c16657-15029	≥84.5	≥90
*ccrB9*	OL880246.1 : 1865–3508	≥84.5	≥90
*ccrB10*	CP007208.1 : 2458421–2460049	≥84.5	≥90
*ccrB11*	LT906460.1:c46380-44755	≥84.5	≥90
*ccrB12*	CP063367.1:c46337-44709	≥84.5	≥90
*ccrC1*	AP006716.1 : 64143–65819	≥84.5	≥90
*ccrC2*	KR187111.1 : 30895–32580	≥84.5	≥90
*ccrC3*	CP065792.1 : 565984–567660	≥84.5	≥90
*ccrC4*	CP059679.1:c1833169-1831493	≥84.5	≥90
*ccrC5*	GU370073.2:c9998-8322	≥84.5	≥90
IS431*	AB033763.2 : 35957–36748	≥95	≥90
IS1272*	AB033763.2 : 28473–30057	≥95	≥90
*mecR1*	X63598.1 : 225–1982	≥95	≥90
*mecR1_2*	FR821779.1 : 37779–39533	≥95	≥90
*mecI*	JF946500.1	≥95	≥90
*mecI_2*	AJ810120.1:c18459-18207	≥95	≥90
*mecI_3*	FR821779.1 : 39530–39904	≥95	≥90
*blaZ*	FR821779.1:c35587-34736	≥95%	≥90%

Novel *mec*: 75–94.9% identity; novel ccr: 70–84.4% identity. *IS431, *IS1272*, *blaZ*, *mecR1* and *mecI* are only included as part of the *mec* gene complex when present on the same contig as the primary *mec* gene and are within 10 Kbp.

Detections are classified into four confidence tiers. For *mec, blaZ* and *IS* genes: *full* (≥95% identity, ≥90% coverage), *partial* (≥95% identity, 75–90% coverage), *novel_full* (75–94.9% identity, ≥90% coverage) and *novel_partial* (75–94.9% identity, 75–90% coverage). For *ccr* genes: *full* (≥84.5% identity, ≥90% coverage) and *novel* (70–84.4% identity). Overlapping blast hits are resolved using a 500 bp threshold and best bitscore selection. The *mec* confirmed threshold (≥95% identity) follows standard practice for allotype-level assignment [22]. The *ccr* confirmed threshold (≥84.5%) was empirically determined: type XI reference *ccrA1* shares 84.9% identity with the *ccrA1* reference sequence, and lowering the threshold from 85.0 to 84.5% was necessary to capture this validated allotype. The default lower bound for novel *ccr* detection is 70%, informed by inter-allotype identity analysis carried out by Huang *et al*. [11]. This can be adjusted via --min-ccr-identity to accommodate detection of highly divergent *ccr* genes, as the IWG-SCC [14] defines novel *ccr* genes down to 50% identity. This threshold was also tested across the 7,297 genomes, and no new hits were detected versus the 70% threshold. A mec_class_notes column in the unified report flags deviations from expected IWG-SCC components for each classified element (e.g. ‘IS431 absent’, ‘mecR1 truncated’), enabling users to assess the completeness of the *mec* complex structure.

‘Novel’ classification is based on these lower limits and indicates that the detected gene shares sufficient homology to be identified as a member of the *mec* or *ccr* gene family but is divergent from all characterized allotypes in the reference database. These may represent genuinely novel allotypes not yet formally described or known allotypes that have accumulated sequence divergence in a particular lineage. Users encountering novel classifications should consider phylogenetic analysis of the detected gene against known allotypes to determine whether it represents a new variant warranting formal characterization.

Detected *ccr* allotype combinations are mapped to IWG-SCC *ccr* complex types 1–22 using a lookup table [11]. Gene-level typing of *mec* allotypes, *ccr* allotypes and *ccr* complex types is the primary output over formal SCC*mec* type assignment (types I–XV) because formal types are generally defined for *S. aureus* SCC*mec* elements, rather than SCC*mec* elements carried by non-*aureus* or *Mammaliicoccus* species. However, for isolates where the gene combination is recognizable, the tool additionally assigns *mec* gene complex class (A–E) based on defining gene combinations near *mecA* [12]: Class A is defined by the presence of *mecI*, Class B by *IS1272* (without *mecI*), Classes C1 and C2 by flanking *IS431* elements (without *mecI* or *IS1272*), with C1 and C2 distinguished by *IS431* orientation (same vs opposite) and Class E by *mecC* with *mecI*. Classes C and D are further distinguished by *IS431* copy number, with Class D carrying a single *IS431* upstream of *mecA*. IS elements, *blaZ*, *mecR1* and *mecI* are only counted as part of the *mec* complex when located within 10 kbp of the primary *mec* gene, on the same contig, preventing false classification from unrelated gene copies elsewhere in the genome. As there are no published identity thresholds for *mec* complex structural genes (*IS431*, *IS1272*, *mecR1*, *mecI*), the same classification thresholds used for *mec* resistance genes were applied (Table 1). The *mec* class and *ccr* complex type combination is then mapped to SCC*mec* types I–XV where a match exists. SCC*mec*Extractor assigns *mec* complex class based on defining functional features rather than requiring all structural components. Class A is assigned when *mecI* is present (indicating intact regulation), regardless of *IS431* status, as IS elements can be lost through deletion while the regulatory architecture remains functional. This approach is more permissive than strict IWG-SCC definitions but avoids failing to classify elements where IS elements have degraded.

These additional classifications are reported alongside gene-level results, enabling both cross-species comparability and formal typing where appropriate. Typing can be applied to extracted SCC elements or to WGSs, enabling gene characterization regardless of extraction outcome.

### Hybrid detection – sccmec-hybrid

To assess similarity to known SCC*mec* types and detect potential hybrid elements, e.g. SCC elements that contain a single *ccr* complex but have sequence similarity to multiple SCC*mec* reference types, a hybrid detection module is available. This module runs blast of each extracted SCC element against 32 bundled SCC*mec* type reference sequences spanning types I–XV (including subtypes IVa–IVn, IIa–IIe, etc). References are used as the blast query against an SCC element database, measuring what proportion of each reference is found within the SCC element.

blast hits are filtered by minimum alignment length (1,000 bp) and minimum percent identity (80%) to remove spurious matches from conserved shared genes (e.g. *mec*, *ccr*). For each reference subtype, the tool calculates reference coverage (percentage of the reference found in the SCC element) and SCC element coverage (where on the SCC element the hits land), with a length-weighted percent identity across all high-scoring segment pairs (HSPs). Subtypes are then grouped into parent types (e.g. IVa–IVn become type IV) by pooling their SCC element footprints and selecting the best subtype by coverage with identity as a tiebreak.

To detect multi-type elements, parent types are ranked by a combined score (SCC element coverage fraction multiplied by weighted identity). The highest-scoring type is designated the primary match and its SCC element footprint is claimed. Each subsequent type is evaluated for novel territory, e.g. regions of the SCC element covered by the secondary type but not by any previously claimed type, using interval subtraction. A type is added as a component if its novel territory exceeds both 1,000 bp and 5% of the SCC element length.

When a typing report from sccmec-type is available (either via the --typing-report flag in standalone mode or automatically in the pipeline), the *ccr* complex count is used to refine classification:

- canonical: single reference type match
- hybrid: multiple reference type matches, single *ccr* complex, indicating potential recombination between SCC*mec* types
- multi_ccr: multiple reference type matches, multiple *ccr* complexes, consistent with a structurally nested/tandem SCC element
- multi_ccr_canonical: single reference type match, multiple *ccr* complexes, a same-type SCC element with duplicated recombinase genes
- no_match: no reference hits above thresholds, a genuinely novel SCC element

Without a typing report, multi-type elements are reported as multi_type (undifferentiated).

In the pipeline, the reference comparison runs as a batch step after all genomes are processed: all extracted SCC element FASTAs are concatenated into a single blast database and queried once, then results are split by SCC element. This reduces the number of blast invocations from one per SCC element to one total. The standalone sccmec-hybrid command accepts extracted SCC element FASTAs and optionally a typing report and also uses batch blast.

Output is a hybrid_summary.tsv with one row per SCC element reporting the best and secondary type matches, their coverage and identity, the classification call, and all component types. An optional --detailed flag produces per-element evidence files listing all reference type matches with their coverage, identity, HSP count and novel territory.

### Unified and summary reporting – sccmec-report

The sccmec-report module merges extraction metadata with typing and hybrid analysis results into a single unified_report.tsv. A priority system prefers gene level typing results obtained from extracted SCC elements, with whole-genome typing as a fallback for genomes where extraction failed. The typing_source column indicates whether typing was performed on the extracted element (sccmec) or the WGS.

The sccmec-report module also produces a sccmec_summary.tsv. This is a user-facing starting point with key results, e.g. *mec* resistance gene, *ccr* complex, SCC*mec* type and hybrid classification. For simplicity, the element_type column indicates whether the result is from an extracted element (SCC*mec* or SCC) or from WGS typing. Users can begin with this file and then explore the unified or individual reports for more detailed information.

### Master pipeline – sccmec-pipeline

For convenience, sccmec-pipeline orchestrates the complete workflow (locate-att, extract, type, hybrid, report) in a single command. It supports batch processing of multiple genomes and both GFF3+FASTA and FASTA-only input modes, with multi-threading.

### Implementation details

SCC*mec*Extractor v1.6.0 is implemented in Python 3.9+with BioPython and blast+ as the only external dependencies. It is available via PyPI (pip instal sccmecextractor), Docker Hub and Singularity. The source code is hosted on GitHub under an MIT licence.

#### Runtime and scalability

Pipeline runtime scales linearly with dataset size. Benchmarked on an Apple M2 Pro laptop (16 GB RAM, 4 threads), the full pipeline (sccmec-locate-att, sccmec-extract, sccmec-type, sccmec-hybrid, sccmec-report) processed 1,454 *S*. *aureus* genomes in ~27 min (~1.1 s genome^−1^), 5,295 non-*aureus Staphylococcus* genomes in ~97 min (~1.1 s genome^−1^) and 548 *Mammaliicoccus* genomes in ~10 min (~1.1 s genome^−1^). The per-genome processing time was consistent across datasets regardless of genome count or species diversity, indicating linear scalability. The reference comparison step uses batch blast (all extracted SCC elements searched in a single invocation) and added less than 2 min even for the largest dataset (828 SCC elements). Peak memory usage was ~130 MB regardless of dataset size, dominated by blast and Python/BioPython overhead rather than input data. The pipeline supports multi-threaded execution via the -t flag; all benchmarks above used four threads. Cross-platform testing on a Windows WSL2 system (AMD Ryzen 9 7940HS, 32 GB RAM, 4 threads) processed the same 1,454 *S*. *aureus* genomes in ~11 min (~0.45 s genome^−1^), confirming portability and efficient performance across operating systems.

## Results

### Typing concordance on known SCC*mec* references

SCC*mec*Extractor was evaluated on 32 reference sequences from the sccmec tool (v1.2.0) test suite, covering SCC*mec* types I–XV including subtypes (Table 2). All 32 references had *ccr* genes correctly detected with correct *ccr* complex type assignment. Of the 28 complete references, *mec* genes were detected in 28/28 and the correct parent SCC*mec* type was assigned for all 28 (100%). Four partial references (IIb, IId, IIe, IVd) lack the *mec* complex region; SCC*mec*Extractor correctly reported no *mec* gene and classified these as not_typeable, while still detecting their *ccr* genes. SCC*mec*Extractor additionally identified tandem/nested SCC elements within three reference sequences: type III (AB037671) contained *ccrC1* beyond the expected *ccrA3/ccrB3*, type IVk (GU122149) carried additional *ccrA1/ccrB1* and type XIV (LC440647) contained *ccrC1* alongside *ccrA4/ccrB4*, all consistent with their previously described structures.

**Table 2.** SCC*mec* type references and typing concordance by SCC*mec*Extractor. SCC*mec*Extractor gene detection results on 32 reference sequences from the sccmec test suite. Four partial references (marked ^P^) correctly show no *mec* detected because their sequences do not include the *mec* complex region. Composite elements (marked ^C^) show additional *ccr* genes beyond the primary type definition

Reference	Accession no.	Expected SCC*mec* type	*ccr* complex	Detected *mec*	Detected *ccr*	*mec* class	Detected SCC*mec* type
Ia	AB033763	Ia	1	*mecA*	*ccrA1+ccrB1*	B	I
Ib	AB433542	Ib	1	*mecA*(partial)	*ccrA1+ccrB1*	B	I
IIa	D86934	IIa	2	*mecA*	*ccrA2+ccrB2*	A	II
IIb^P^	AB127982	IIb	2	–	*ccrA2+ccrB2*	–	nt
IIc	AJ810120	IIc	2	*mecA*	*ccrA2+ccrB2*	A	II
IId^P^	AB261975	IId	2	–	*ccrA2+ccrB2*	–	nt
IIe^P^	AB435013	IIe	2	–	*ccrA2+ccrB2*	–	nt
III^C^	AB037671	III	3	*mecA*	*ccrA3+ccrB3+ccrC1*	A	III
IVa	AB063172	IVa	2	*mecA*	*ccrA2+ccrB2*	B	IV
IVb	AB063173	IVb	2	*mecA*	*ccrA2+ccrB2*	B	IV
IVc	AB096217	IVc	2	*mecA*	*ccrA2+ccrB2*	B	IV
IVd^P^	AB097677	IVd	2	–	*ccrA2+ccrB2*	–	nt
IVg	DQ106887	IVg	2	*mecA*	*ccrA2+ccrB2*	B	IV
IVi	AB425823	IVi	2	*mecA*	*ccrA2+ccrB2*	B	IV
IVj	AB425824	IVj	2	*mecA*	*ccrA2+ccrB2*	B	IV
IVk^C^	GU122149	IVk	2	*mecA*	*ccrA1+ccrA2+ccrB1+ccrB2*	B	IV
IVl	AB633329	IVl	2	*mecA*	*ccrA2+ccrB2*	B	IV
IVm	AB872254	IVm	2	*mecA*	*ccrA2+ccrB2*	B	IV
IVn	KX385846)	IVn	2	*mecA*	*ccrA2+ccrB2*	B	IV
Va	AB121219	Va	5	*mecA*	*ccrC1*	C2	V
Vb^C^	AB512767	Vb	5	*mecA*	*ccrC1*×2	C2	V
Vc^C^	AB505629	Vc	5	*mecA*	*ccrC1*×2	C2	V
VI	AF411935	VI	4	*mecA*	*ccrA4+ccrB4*	B	VI
VII	AB373032	VII	5	*mecA*	*ccrC1*	C1	VII
VIII	FJ390057	VIII	4	*mecA*	*ccrA4+ccrB4*	A	VIII
IX	AB505628	IX	1	*mecA*	*ccrA1+ccrB1*	C2	IX
X	AB505630	X	7	*mecA*	*ccrA1+ccrB6*	C1	X
XI	FR821779	XI	8	*mecC*	*ccrA1+ccrB3*	E	XI
XII	KR187111	XII	9	*mecA*	*ccrC2*	C2	XII
XIII	MG674089	XIII	9	*mecA*	*ccrC2*	A	XIII
XIV^C^	LC440647	XIV	4;5	*mecA*	*ccrC1+ccrA4+ccrB4*	A	XIV
XV	NZ_CP080249	XV	7	*mecA*	*ccrA1+ccrB6*	A	XV

SCC*mec*Extractor assigns parent SCC*mec* types (I–XV); subtype assignment (e.g. IVa) requires whole-cassette reference comparison, reported separately in the hybrid detection output. P Partial/incomplete reference sequence; CComposite element with additional *ccr* genes. nt refers to not_typeable due to partial sequences.

Both SCC*mec*Extractor and sccmec correctly assigned the parent SCC*mec* type for all 28 complete references. sccmec reported cross-subtype ambiguity for four references, including one (IVk) where ambiguity crossed type boundaries (types IV and VI); SCC*mec*Extractor did not produce these ambiguities. For the four partial references, sccmec’s whole-cassette matching correctly identified the subtype despite the absence of *mecA*, whereas SCC*mec*Extractor’s gene-level approach requires *mec* class assignment for type determination. The hybrid analysis module confirmed all 32 as canonical single-type elements with no false hybrid calls.

### Typing concordance on *S. aureus* whole-genome sequences

To validate typing accuracy at scale, SCC*mec*Extractor was compared against sccmec on 1,454 *S*. *aureus* complete genomes from NCBI (Table S1, available in the online Supplementary Material). *S. aureus* genomes were obtained by using datasets [23] with --assembly-level ‘complete’ or ‘chromosome’, with the taxon flag ‘*Staphylococcus aureus*’ to ensure contiguous sequence data suitable for SCC*mec* boundary resolution. No filtering was applied based on resistance phenotype, isolation source or any other biological criterion, although genome GCF_020167475.1 was excluded because it was misclassified as complete and contained 102 contigs. However, complete genome assemblies in public databases are enriched for clinically significant strains, which may inflate the proportion of SCC*mec*-carrying genomes relative to the species as a whole.

Within this dataset, both tools detected *mecA*/*mecC* in the same 653 genomes (100% concordance) and agreed on *ccr* complex type assignment for all 606 genomes where sccmec assigned a single type (100% concordance). An additional 21 genomes assigned ‘multiple’ by sccmec also matched SCC*mec*Extractor at the gene level. SCC*mec*Extractor and sccmec agreed on SCC*mec* type assignment, for those genomes in which a clear type could be assigned, 606 of 639 typed genomes (94.8%), with 586 exact matches and 20 where both tools reported multiple types. Eight SCC*mec* type disagreements involved composite elements carrying multiple *ccr* complexes, where the tools differed in primary *ccr* selection: SCC*mec*Extractor selects the *ccr* gene closest to the primary *mec* gene, while sccmec’s selection is implicit in its typing schema. SCC*mec*Extractor assigned types for three genomes where sccmec could not resolve targets to a type due to one genome missing *ccrA4* from sccmec’s detection and two genomes having *IS431* or *IS1272* below sccmec’s 90% identity threshold. In contrast, sccmec’s whole-cassette matching assigned two genomes as type XII that SCC*mec*Extractor could not type due to *ccrC2* residing outside the extracted element boundary. Notably, SCC*mec*Extractor distinguishes *mec* class C1 from C2 based on *IS431* orientation, enabling the type VII versus type V distinction that sccmec’s target-based approach cannot resolve. For standalone gene-level typing (sccmec-type, single-threaded), runtime was ~2.1 s/genome for SCC*mec*Extractor versus 2.6 s genome^−1^ for sccmec on this dataset. The runtime comparison is for gene-level typing only, as this is the comparable function between the two tools.

The two tools take complementary approaches that diverge for cases beyond standard *S. aureus* SCC*mec* types. sccmec assigns formal SCC*mec* types (I–XV) by matching genome sequences against whole-cassette reference regions, an approach well-suited to epidemiological typing of *S. aureus*. SCC*mec*Extractor takes a gene-level approach, reporting individual *mec* and *ccr* allotypes, their *ccr* complex type, *mec* class and determining SCC*mec* type (I–XV) where the combination matches a defined type. This provides a consistent framework that extends to novel gene combinations and non-*aureus* species.

Beyond type assignment concordance, the gene-level approaches reveal additional differences. First, SCC*mec*Extractor detected *ccr* genes in 18 genomes carrying novel allotypes (*ccrA5*, *ccrA6*, *ccrA9*, *ccrA11*, *ccrB5*, *ccrB12*, *ccrC4*) absent from sccmec‘s targets reference database. Second, SCC*mec*Extractor identified 89 genomes (12.3% of *ccr*-positive) carrying non-*mec* SCC elements, of which sccmec correctly detected their *ccr* genes in its targets output but could not assign a type without a *mec* gene, reflecting its design focus on SCC*mec* typing rather than broader SCC element diversity. Third, among *mecC*-carrying genomes, SCC*mec*Extractor distinguished specific allotypes (*mecC*, *mecC1*), whereas sccmec detected all as ‘*mecA*+’ as sccmec reports a binary ‘*mec*’ presence column that does not distinguish *mecA* from *mecC* variants. This is sufficient for flagging the presence of a *mec* gene but not for distinguishing *mec* gene variants, which is relevant given the reduced affinity of *mecC* for almost all β-lactam antimicrobials [24].

The approaches also differ in their handling of SCC elements carrying multiple *ccr* complexes. Multiple *ccr* complex types within a single genome can indicate either a tandem or nested arrangement of distinct SCC elements or a single element that carries multiple *ccr* genes, as seen in some defined SCC*mec* types (e.g. type XII carries both *ccr* complex 9 and *ccrA1*). SCC*mec*Extractor distinguishes these by requiring structural evidence: an SCC element is only classified as composite when internal *att* site boundaries separate the *ccr* complexes into distinct regions flanked by their own *att* site pairs (Fig. 2).

This resulted in five composite SCC element extractions out of 72 genomes carrying multiple *ccr* complex types, representing 0.8% of the total extracted SCC elements for *S. aureus,* with the remaining 67 carrying multiple *ccr* complexes within a single element. In contrast, sccmec flagged 105 genomes with a ‘multiple’ subtype, indicating matches to more than one reference cassette. Exploring this further reveals three distinct situations: 67 (64%) were subtype ambiguity within a single type (e.g. simultaneous IVa and IVn matches to a single element); 18 (17%) reflected partial sequence similarity to a second reference cassette despite carrying only one *ccr* complex; and 20 (19%) carried genuinely distinct *ccr* complex types. SCC*mec*Extractor’s gene-level approach identified 72 genomes carrying multiple *ccr* complex types, providing a more complete picture than sccmec’s 20 multi-type calls, which represent only those genomes matching multiple distinct reference cassettes. Overall, these results illustrate how whole-cassette reference matching and *att* site-based boundary detection address complementary aspects of SCC biology.

### SCC element extraction across staphylococci and *Mammaliicoccus*

The full SCC*mec*Extractor pipeline was applied to three datasets (Table S1), all using blast-based *rlmH* detection in FASTA-only mode and FASTA+GFF3 mode, although for benchmarking the blast-based results were used. The datasets included 1,454 *S*. *aureus* complete genomes, 5,295 non-*aureus Staphylococcus* genomes from 63 species and 548 *Mammaliicoccus* genomes from 6 species. Extraction performance is summarized in Table 3. As with the *S. aureus* dataset, non-*aureus* staphylococci and *Mammaliicoccus* genomes were obtained using datasets.

**Table 3.** SCC element extraction performance across three datasets. Summary of SCC element extraction using the full SCC*mec*Extractor pipeline across *S. aureus*, non-*aureus Staphylococcus* and *Mammaliicoccus*

Metric	*S. aureus*	Non-aureus *Staphylococcus*	*Mammaliicoccus*	Combined
Total genomes	1,454	5,295	548	7,297
Species	1	63	6	70
*ccr*-positive genomes	726 (49.9%)	2918 (55.1%)	269 (49.1%)	3913 (53.6%)
**Total extracted**	**630** (**43.3%**)	**828** (**15.6%**)	**104** (**19.0%**)	**1,562** (**21.4%**)
*ccr*+extraction rate	86.8%	28.3%	38.6%	39.9%
Effective extraction rate	87.3%	58.8%	61.9%	–
**Element classification**				
SCC*mec* (*mec+ccr*)	553 (87.8%)	368 (44.4%)	25 (24.0%)	946 (60.6%)
Non-*mec* SCC (*ccr*, no *mec*)	77 (12.2%)	460 (55.6%)	79 (76.0%)	616 (39.4%)
**Features**				
Composite extractions	5 (0.8%)	76 (9.2%)	11 (10.6%)	92 (5.9%)
Fallback extractions	11 (1.7%)	37 (4.5%)	12 (11.5%)	60 (3.8%)
No-*ccr* elements (SCI)	94	284	72	450
Origin-spanning detected	2	3	0	5
**Failure analysis (*ccr*+**)				
Assembly-limited failures	2	1,452 (69.5%)	100 (60.6%)	1,554
Right_only (no *attL*)	66	477	49	592
Addressable failures	80	545	60	685
Median (bp)	29,415	31,557	37,326	–
Range (bp)	12,109–99,888	11,153–158,206	14,630–107,704	–

Effective extraction rate excludes assembly-limited genomes (cross-contig, origin-spanning, *attL*/*rlmH* on different contigs) and *ccr* genes without a valid pair e.g. lone *ccrA* without a corresponding *ccrB*. right_only counts include *attB* overlap artefacts (31 non-*aureus*, 9 *Mammaliicoccus*) where the *attL* regex matched the empty chromosomal integration site rather than a genuine element boundary.

To distinguish tool performance from assembly limitations, we report two extraction rates: the *ccr*-positive extraction rate and the effective extraction rate. The *ccr*-positive rate measures extraction success among all *ccr*-positive genomes; the effective rate excludes genomes where extraction is impossible regardless of tool capability (see Implementation extraction diagnostics). Across all three datasets, 1,562 SCC elements were extracted from 7,297 genomes spanning 70 species and 2 genera (Table S3). The extraction rate among *ccr*-positive genomes was 86.8% for *S. aureus*, 28.3% for non-*aureus Staphylococcus* and 38.6% for *Mammaliicoccus*. The lower non-*aureus* and *Mammaliicoccus* rates reflect the predominance of draft assemblies. To assess tool performance independent of assembly quality, we calculated an effective extraction rate by excluding genomes where extraction is impossible regardless of tool capability: assembly-limited genomes (those with *att* sites on different contigs, origin-spanning or *attL*/*rlmH* on different contigs) and genomes with unpaired *ccr* genes (lone *ccrA* or *ccrB* without a functional recombinase pair). The effective extraction rate was 87.3% (630/722) for *S. aureus*, 58.8% (828/1,408) for non-*aureus Staphylococcus* and 61.9% (104/168) for *Mammaliicoccus*. Filtering the extraction results based on species revealed some species within the non-*aureus* dataset had effective extraction rates of 100.0%, with clinically relevant species such as *Staphylococcus saprophyticus, Staphylococcus lugdunensis* and *Staphylococcus pseudintermedius* having rates of 94.9, 92.9 and 70.9%, respectively (Table S4). In contrast, some clinically relevant species had lower effective extraction rates than the average, e.g. *S. epidermidis* 57.5%, or slightly higher as in the case of *Staphylococcus haemolyticus* at 62.9%.

#### Failure analysis

Assembly fragmentation was the dominant cause of extraction failure across all datasets. For *S. aureus* (all complete genomes), cross-contig failures were absent; the 96 *ccr*-positive non-extracted genomes comprised 66 right_only (no matching *attL* pattern), 12 classified as no_ccr_element (*ccr* detected outside element bounds), 9 left_only (no *ccr* genes found between *attL* and *rlmH*), 4 with no *att* sites detected, 2 origin_spanning genomes (flagged with wrap-around coordinates), 2 with unpaired *ccr* genes (lone *ccrA* without a *ccrB* partner) and 1 element size_out_of_range. For non-*aureus Staphylococcus*, 1,452 of 2,090 *ccr*-positive failures (69.5%) were assembly-limited: 1,270 cross-contig, 175 with *attL* and *rlmH* on different contigs, 4 with no *rlmH* detected (due to the gene being split over two contigs) and 3 origin-spanning. The remaining 545 addressable failures included 477 genomes requiring novel *attL* patterns, e.g. right_only failures, where extreme bridge sequence diversity (45+unique bridges) yielded diminishing returns for further pattern expansion. Another 33 with size_out_of_range, 25 with no *att* sites detected and 10 left_only. For *Mammaliicoccus*, 100 of 165 *ccr*-positive failures (60.6%) were assembly-limited (93 cross-contig, 6 with *attL* and *rlmH* on different contigs, 1 with no *rlmH* detected as the gene was split between two contigs), leaving 60 addressable failures including 49 right_only.

#### Composite elements

Composite elements were detected in 5 (0.8%) of *S. aureus* extractions, 76 (9.2%) of non-*aureus* and 11 (10.6%) of *Mammaliicoccus* extractions.

#### Fallback extraction

SCC elements were recovered for 11 *S*. *aureus* (1.7% of extractions), 37 non-*aureus* (4.5%) and 12 *Mammaliicoccus* (11.5%) genomes. The increasing fallback rate across datasets reflects growing *attR* site divergence away from the *Staphylococcus*-derived patterns.

#### Fallback extraction reliability

Fallback extractions, where the right boundary is inferred from the *rlmH* gene position rather than an explicit *attR* match, accounted for 1.7–11.5% of extractions across datasets. Three constraints were included to mitigate false positives: *rlmH* must be on the same contig as *attL*, the distance must not exceed 120 kbp and *ccr* genes must be present between the two boundaries, e.g. *rlmH* and *attL*. These SCC elements are flagged as fallback_extracted in the unified report, enabling users to distinguish them from standard extractions. For downstream analysis, we recommend that users treat fallback extractions as lower confidence and, where possible, manually inspect the 3′ end of *rlmH* to identify why *attR* was not detected. Some causes include deviations from the conserved core motif (TATCATAA), such as loss of the central cytosine required for recombination, or novel *attR* sequence variants not yet represented in the tool’s pattern set.

#### No-*ccr* elements

The *ccr* gene check correctly classified 450 *att* site pairs across all datasets as no_ccr_element (SCI): 94 in *S. aureus*, 284 in non-*aureus Staphylococcus* and 72 in *Mammaliicoccus*. In *Mammaliicoccus*, 63 of 72 SCI elements (87.5%) carried *mec* genes (predominantly *mecA1*), consistent with the established role of *Mammaliicoccus sciuri* group species as the evolutionary reservoir of *mecA*-family genes [25], where native *mec* homologues are chromosomally encoded independently of SCC*mec*.

### SCC element diversity beyond SCC*mec*

A striking gradient in SCC element composition emerged across the three datasets (Table 3). In *S. aureus*, 87.8% (553/630) of extracted elements were SCC*mec* (carrying both *mec* and *ccr*) and only 12.2% (77/630) were non-*mec* SCC. In non-*aureus Staphylococcus*, there were more non-*mec* SCC elements, at 55.6% (460/828) vs 44.4% (368/828). In *Mammaliicoccus*, non-*mec* SCC dominated at 76.0% (79/104), the highest proportion within our datasets.

This gradient reflects both MRSA-centric bias in *S. aureus* research and the genuine prevalence of non-*mec* SCC elements outside *S. aureus*. Among the 2,918 *ccr*-positive non-*aureus* genomes, 1,295 (44.4%) of those genomes carried *ccr* without any detectable *mec* gene. Extracted non-*mec* SCC carriage varied dramatically by species: environmental and commensal species such as *Staphylococcus equorum*, *Staphylococcus chromogenes*, *Staphylococcus xylosus* carried predominantly non-*mec* SCC (100%), while clinical species such as *S. epidermidis* (42.2% non-*mec* SCC) and *S. pseudintermedius* (7.6% non-*mec* SCC) carried mostly SCC*mec* (Table S5).

#### Dataset composition and interpretive caveats

Although no resistance-based filtering was applied during genome selection, the *S. aureus* complete genome set available in public databases is inherently enriched for clinically significant isolates, predominantly MRSA. Examining the metadata for the three datasets reveals that 74.9% of the *S. aureus* genomes are human-derived, reflecting the clinical sampling bias. The non-*aureus* and *Mammaliicoccus* datasets were more evenly distributed across isolation sources (non-*aureus*: 62.5% human, 18.1% animal; *Mammaliicoccus*: 33.4% human, 28.1% animal, 13.5% environmental), and their higher non-*mec* SCC prevalence (55.6 and 76.0%) is more likely to reflect genuine SCC diversity. Therefore, the proportion of SCC*mec* among extracted *S. aureus* elements (87.8%) should not be interpreted as a species-wide estimate but reflects the clinical enrichment within the publicly available complete genome dataset. These biases affect the biological prevalence estimates but not tool accuracy, as typing concordance and extraction logic are independent of dataset composition.

#### Novel *ccr* diversity

In non-*aureus Staphylococcus*, 110 genomes (3.8%) carried novel *ccr* allotypes below the 84.5% identity threshold for confirmed assignment. For *Mammaliicoccus*, novel *ccr* diversity was far greater: 46.8% of *ccr*-positive genomes carried *ccr* allotype combinations not represented in the current IWG-SCC complex type scheme (novel_combination), with *ccr* complex types 20 (*ccrA9/ccrB5*) and 21 (*ccrC4*) being the most common defined types, types rare or absent in the *S. aureus* dataset.

#### *Mammaliicoccus* gene carriage

The *Mammaliicoccus* gene carriage pattern differed markedly from *Staphylococcus. mecA1* (not *mecA*) was the dominant *mec* gene, detected in 321/548 genomes (58.6%), supporting the hypothesis that *M. sciuri* group species represent the evolutionary origin of *mecA*. Strikingly, 42.2% of genomes carried *mec* without detectable *ccr*, suggesting widespread chromosomal *mec* integration outside SCC element context.

### Hybrid element detection

To validate the reference comparison module, it was first applied to the 32 SCC*mec* type reference sequences listed in Table 2. All 32 were correctly classified as canonical, confirming that each reference matches only its own type with no false hybrid calls.

The module was then applied to all 1,562 extracted SCC elements across the three datasets (Table 4). Elements were classified into five categories based on the number of reference types matched and the *ccr* complex count from gene-level typing.

**Table 4.** Hybrid classification across three datasets

Category	Definition	*S. aureus* (*n*=630)	Non-*aureus Staphylococcus* (*n*=828)	*Mammaliicoccus* (*n*=104)	Combined (*n*=1,562)
Canonical	Single type, single *ccr*	485 (77.0%)	298 (36.0%)	26 (25.0%)	809 (51.8%)
Hybrid	Multi-type, single *ccr*	95 (15.1%)	354 (42.8%)	74 (71.2%)	523 (33.5%)
Multi_ccr	Multi-type, multiple *ccr*	28 (4.4%)	158 (19.1%)	4 (3.8%)	190 (12.2%)
Multi_ccr_canonical	Single type, multiple *ccr*	22 (3.5%)	9 (1.1%)	0 (0.0%)	31 (2.0%)
No_match	No reference hits	0.0%	9 (1.1%)	0 (0.0%)	9 (0.6%)

Classification uses both blast-based reference comparison and *ccr* complex count from gene-level typing.

A clear gradient in canonical element prevalence was observed across datasets. In *S. aureus*, 77.0% of elements matched a single reference type (canonical), compared with 36.0% in non-*aureus Staphylococcus* and 25.0% in *Mammaliicoccus*. Conversely, the hybrid rate, those elements matching multiple reference types but carrying only a single *ccr* complex, increased from 15.1% in *S. aureus* to 42.8% in non-*aureus* and 71.2% in *Mammaliicoccus*. This gradient likely reflects the increasing evolutionary distance from the *S. aureus*-derived reference set and highlights the need for formally characterized non-*aureus* SCC*mec* reference types to distinguish genuine hybrid elements from lineages that are simply unrepresented in the current typing scheme.

The most frequent hybrid combination in non-*aureus Staphylococcus* was III with XV (92 elements), representing ~25 kbp elements carrying *ccr* complex 3 (typed as SCC*mec* III). These elements covered only 33% of the 68 kbp type III reference but shared 60% coverage with the smaller 35 kbp type XV reference, suggesting that they carry a conserved subset of type III sequence supplemented by regions more like type XV. In *Mammaliicoccus*, III with XV (nine elements) and III with IV (eight elements) were the most common combinations.

multi-ccr elements carrying multiple *ccr* complexes with multi-type similarity were found in 28 *S. aureus* (4.4%), 158 non-*aureus* (19.1%) and 4 *Mammaliicoccus* (3.8%) elements. While multi_ccr_canonical, those elements with multiple *ccr* complexes matching only a single reference type, was identified in 22 *S. aureus* and 9 non-*aureus* elements. All 22 *S. aureus* cases were type III elements carrying *ccr* complexes 3 and 5, as to be expected as this SCC*mec* reference type carries a SCC*Hg* region encoding for *ccrC1*, or *ccr* complex 5.

Nine non-*aureus* elements produced no reference hits above the detection thresholds (no_match). All nine were non-*mec* SCC elements carrying unusual *ccr* combinations (*ccrC1*, *ccrC4* or *ccrA10*/*ccrB10*) and were classified as not_typeable by gene-level typing. These represent genuinely novel SCC elements with no significant sequence similarity to any characterized SCC*mec* type.

### Comparison with existing tools

Table 5 summarizes the feature comparison between SCC*mec*Extractor and existing tools. Typing concordance with sccmec was demonstrated on both reference sequences (32/32 correct *ccr* complex types) and *S. aureus* whole genomes (100% gene-level concordance and 94.8% SCC*mec* type concordance on 1,454 genomes). As SCC*mec*Finder and SCCmec_CLA could not be installed (see below), the comparison in Table 5 is based on published descriptions, documentation and source code review rather than direct benchmarking.

**Table 5.** Feature comparison with existing SCC*mec* tools

Feature	SCC*mec*Extractor	sccmec (Petit III)	SCCmecFinder (CGE)	SCCmec_CLA
**Status**	Active	Active	Non-functional*	Non-reproducible†
**Element extraction**	Yes	No	No	Yes
**Non-*mec* SCC extraction**	Yes	No	No	No
***att* site detection**	20 patterns (*attR+attL*)	No	No	Distal only (8 bp core+blast)‡
**Composite detection**	Yes	No	No	No
**Gene-level typing**	*mec+ccr* allotypes	Formal types I–XV	Types I–XI (web)	Types I–XI (synteny+kNN)
**Novel gene detection**	Yes	Via region comparison	No	Via MASH distance
**Extraction diagnostics**	Yes (categorized failures)	n/a	n/a	No
**FASTA-only input**	Yes (blast *rlmH*)	Yes	Yes	Yes (runs Prokka)
**Installation**	pip / Docker / Singularity	conda	Manual	Manual
**Dependencies**	BioPython, blast+	camlhmp	Python 2, BioPerl, legacy blast	Prokka, MASH, BioPerl, scikit-learn
**Platform**	macOS+Linux	macOS+Linux	Linux	Linux only
**Python**	3.9+	Current	2.7	3.7
**Licence**	MIT	MIT	MIT	MIT

Feature comparison based on published descriptions, documentation and source code review. Direct benchmarking was only possible with sccmec (Petit III).

*Requires Python 2.7, legacy blast (blastall/formatdb), missing reference databases and BioPerl. Web server (v1.2) operational but limited.

†installation fails with undocumented Perl XML::Simple dependency and outdated matplotlib 2.0.2; last updated 7 years ago.

‡Assumes the last 60 bp of *orfX* represents *attR*, does not search for *attR* patterns.

SCC*mec*Finder (CGE) could not be evaluated as a standalone tool. The software requires Python 2.7, legacy blast (blastall/formatdb), gene databases not included in the download and BioPerl; it is effectively non-functional for local benchmarking. The CGE web server (v1.2) remains operational but is limited to SCC*mec* types I–XI and does not perform extraction.

SCCmec_CLA could not be evaluated as installation failed on a standard Linux HPC following the repository’s instructions, with two independent errors: an undocumented Perl XML::Simple dependency required by the bundled PROKKA 1.12, as well as an outdated matplotlib 2.0.2 requirement that fails to compile without system-level library access. The tool was last updated 7 years ago, uses Python 3.7 and bundles a Linux-only Mash binary. However, reviewing the code confirmed that it locates the distal *att* (referred to here as *attL* but by SCCmec_CLA as *attR*) site boundary using the 8 bp core motif of the *att* sequences, with blast validation against a reference database of known *att* sites. The approximation of the *attR* (referred to as *attL* by SCCmec_CLA) within *rlmH* is reasonable, although this results in no specific *attR* sequence being reported. Finally, the element typing is a thorough approach, examining synteny of the *mec* complex, identifying *ccr* allotypes and using k-nearest-neighbour subtype prediction against reference cassettes; however, typing only occurs on extracted SCC element sequences, therefore if extraction fails no WGS typing occurs.

## Discussion

SCC*mec*Extractor fills a bioinformatic gap for staphylococcal genomics by combining SCC element extraction, gene-level typing and non-*mec* SCC detection in a single package. Benchmarking across 7,297 genomes from 70 species and 2 genera demonstrates that it performs accurately and reveals biological diversity invisible to existing tools.

### Automated non-*mec* SCC detection is a biologically significant capability

The progressive increase in non-*mec* SCC prevalence from *S. aureus* (12.2%) to non-*aureus Staphylococcus* (55.6%) to *Mammaliicoccus* (76.0%) reveals that non-*mec* SCC elements are the dominant SCC class outside *S. aureus*. These SCC elements carry *ccr* recombinases and integrate at *rlmH* but lack methicillin resistance genes. Although individual non-*mec* SCC elements have been characterized and named (e.g. SCC*Hg,* SCC*cap1*, SCC*fur*), no systematic survey across species has been possible because existing tools cannot detect them. Their prevalence, particularly in environmental and commensal species, suggests that they play important roles in staphylococcal adaptation beyond antibiotic resistance. The extraction of 616 non-*mec* SCC sequences across this study provides material for downstream functional analysis.

### Gene-level typing is taxonomically appropriate for cross-species work

Formal SCC*mec* types I–XV are defined by IWG-SCC specifically for *S. aureus* and may not capture the diversity of SCC elements in other species, where novel *ccr* allotype combinations and *mec* complex structures are common. Gene-level reporting of *mec* allotypes, *ccr* allotypes and *ccr* complex types provides a consistent framework for characterizing SCC elements regardless of host species. SCC*mec*Extractor assigns formal SCC*mec* types where the gene combination matches a defined type, but gene-level results remain the primary output to ensure cross-species applicability.

### Cross-genus applicability extends the tool beyond *Staphylococcus*

The successful extraction of 104 SCC elements from *Mammaliicoccus* (formerly the *S. sciuri* group [26]) demonstrates that SCC*mec*Extractor can be applied to related genera with the addition of genus-specific *rlmH* sequences to the reference database. The *Mammaliicoccus* analysis revealed that *mecA1*, not *mecA* is the dominant *mec* gene in this genus and that nearly half of *ccr*-positive genomes carry novel *ccr* complex types not represented in existing typing schemes. These findings support the use of genus-wide SCC element analysis for understanding the evolutionary origins and diversity of SCC elements.

### Composite element detection reveals tandem SCC integration across species

Across all datasets, 92 of 1,562 extracted SCC elements (5.9%) were confirmed composites, where internal *att* site boundaries separated distinct SCC cassettes within a single extracted SCC element, spanning 20 species across both genera. Composite prevalence increased outside *S. aureus* (0.8%) to non-*aureus* staphylococci (9.2%) and *Mammaliicoccus* (10.6%), mirroring the broader trend of greater SCC element diversity in non-*aureus* species. The composition of these composite elements also followed this pattern: all 5 *S. aureus* composites were SCC*mec*, while 59.2% of non-*aureus* composites (45/76) were non-*mec* SCC. Composite confirmation requires identification of internal *att* site boundaries and verification of *ccr* gene content in the outer region, a capability unique to extraction-based approaches. Reference-matching tools can identify genomes with multiple *ccr* complex types but cannot distinguish genuine composite element integration from multi-*ccr* single elements or subtype ambiguity.

The practical implications of composite SCC elements are present for both epidemiology and evolutionary analysis. As they carry multiple recombinase systems and intact *att* sites, they can potentially excise as different structural units depending on which *ccr* complex is active [27, 28], which can complicate our understanding of the movement of these elements. It is also unknown whether different recombinases within a composite can act on *att* sites generated by other *ccr* complexes, which could enable excision of the entire composite as a single unit, or excision of a non-cognate inner element by the outer element’s recombinases. The composite structure also records integration chronology, with the assumption that the outermost element, i.e. furthest from *rlmH,* was integrated first and pushed downstream upon the second integration event, enabling reconstruction of the sequence of SCC element acquisition events within a lineage. This underscores the importance of distinguishing composite SCC elements from hybrid SCC elements, i.e. those with a single *ccr* complex but nucleotide identity shared between SCC*mec* types.

### Hybrid detection distinguishes hybrid from multi-*ccr* SCC elements

The hybrid detection module provides a complementary perspective to gene-level typing by assessing whole-element similarity to characterized SCC*mec* types. Classifying SCC elements by both the number of reference type matches and the number of *ccr* complexes revealed four distinct categories with different biological implications. Hybrid SCC elements (single *ccr*, multi-type similarity) may represent genuine inter-type recombination events, where portions of the SCC element derive from different SCC*mec* lineages while retaining a single recombinase system. multi-ccr elements (multiple *ccr*, multi-type similarity) are consistent with tandem or nested cassette arrangements from different lineages, although without internal *att* site boundaries these cannot be confirmed as composites by SCC*mec*Extractor. The multi_ccr_canonical category, a single-type match despite multiple *ccr* complexes, suggests duplication of a same-type element. The decreasing canonical rate from *S. aureus* (77.0%) through non-*aureus Staphylococcus* (36.0%) to *Mammaliicoccus* (25.0%) should be interpreted cautiously: because all 32 reference sequences are derived from *S. aureus*, SCC elements from other species may appear as hybrids when they are simply divergent from the reference set rather than products of inter-type recombination. The hybrid analysis capability of SCC*mec*Extractor can be used to examine this, for non-*aureus* and *Mammaliicoccus* SCC elements. Those SCC elements that share the same hybrid similarity pattern can be compared to each other, which may reveal circulating ‘canonical’ elements within these groups. The nine no_match SCC elements, all carrying unusual *ccr* combinations absent from the typing scheme, underscore SCC element diversity beyond current reference coverage.

### Assembly quality is the primary extraction limitation

The same-contig requirement, mandating that both *att* sites reside on a single contig, is a deliberate design choice that prioritizes extraction confidence. Assembly fragmentation accounted for 69.5% of *ccr*-positive failures in non-*aureus* staphylococci and 60.6% in *Mammaliicoccus*. This is a field-wide challenge inherent to short-read draft assemblies, not a limitation specific to SCC*mec*Extractor. Cross-referencing *ccr* gene locations with *att* site coordinates revealed that an additional 39 non-*aureus* genomes classified as left_only failures had *ccr* genes co-located with *attL* sites on a different contig from *rlmH*, suggesting the true proportion of assembly-limited failures is higher than the current classification captures. The *ccr* and *mec* gene location columns in the typing output facilitate manual review of such ambiguous cases. Long-read sequencing technologies that produce more contiguous assemblies would substantially improve extraction rates. SCCmec_CLA addresses this by concatenating contigs, but this approach introduces uncertainty about SCC element integrity that we consider unacceptable for confident downstream analysis. Our strategy is to supply boundary locations, enabling users to bridge contig gaps via targeted approaches such as PCR and Sanger sequencing.

### Guidance on input genome quality

Complete or closed genome assemblies will yield the highest extraction rates, as demonstrated by the 86.8% *ccr*-positive extraction rate on *S. aureus* complete genomes. For draft assemblies, extraction success depends not on global assembly metrics (e.g. N50) but on whether both *att* sites of a given SCC element are captured on a single contig, a property that cannot be predicted from summary statistics alone. Users should therefore not interpret extraction failure as absence of an SCC element. The ambiguous_att_sites.tsv output reports detected *att* site coordinates, contig locations and failure reasons for all non-extracted genomes, enabling users to identify cases where primer walking or long-read sequencing could close a specific gap and recover the SCC element. For surveillance applications where extraction is not required, sccmec-type can characterize *mec* and *ccr* gene content, *mec* complex class and predicted SCC*mec* type directly from any assembly, as gene-level typing does not depend on an extracted SCC element.

### FASTA-only mode removes annotation dependency

The blast-based *rlmH* detection mode, validated against a 70-species reference database, enables SCC*mec*Extractor to process unannotated assemblies without requiring Bakta, Prokka or other annotation tools. This simplifies integration into routine genomic surveillance pipelines. Furthermore, users can supply their own reference *rlmH* genes for diverse isolates not covered by SCC*mec*Extractor *rlmH* database.

## Limitations

SCC*mec*Extractor has several limitations. First, the same-contig requirement means that SCC elements spanning contig boundaries cannot be extracted; this is the primary constraint for draft assemblies. The ambiguous_att_sites.tsv output provides per-genome *att* site coordinates and failure categorization to facilitate manual investigation of these cases. Second, the tool requires *rlmH* to anchor the *attR* site; the 70-species reference database covers the genus well, but novel or highly divergent species may require a custom reference via the --rlmh-ref option. Genome annotation would overcome this limitation. Third, the current 20 *att* site patterns cover the majority of known SCC elements, but extreme *attL* bridge diversity across non-*aureus* species (45+ unique bridges identified) means some species-specific variants remain unrepresented. Fourth, *SCCmec*Extractor does not perform J region subtyping. Fifth, the reference comparison module uses 32 SCC*mec* type reference sequences derived from *S. aureus*. SCC elements from non-*aureus* species classified as hybrid should be interpreted as having similarity to multiple *S. aureus*-derived references, which may reflect genuine inter-type recombination, evolutionary divergence from the reference set, or the existence of non-*aureus* SCC element lineages not represented by the current reference types.

## Future directions

Planned extensions include expanded *att* site patterns as more SCC element diversity is characterized through long-read sequencing, expansion of the SCC*mec* type reference database as new types are formally defined and support for user-contributed reference types for hybrid detection.

## Supplementary material

10.1099/mgen.0.001809 Supplementary Material 1.

## Abbreviations

CGE: Center for Genomic Epidemiology
HSP: high-scoring segment pairs
IS: insertion sequence
IWG-SCC: International Working Group on the Classification of Staphylococcal Cassette Chromosome Elements
J: Joining Regions of SCC elements
MRSA: methicillin-resistant *Staphylococcus aureus*
SCC: staphylococcal cassette chromosome
SCCcap1: Capsule biosynthesis encoding SCC element
SCCHg: Mecury Resistance encoding SCC element
SCC*mec*: staphylococcal cassette chromosome *mec*
WGS: whole-genome sequence
